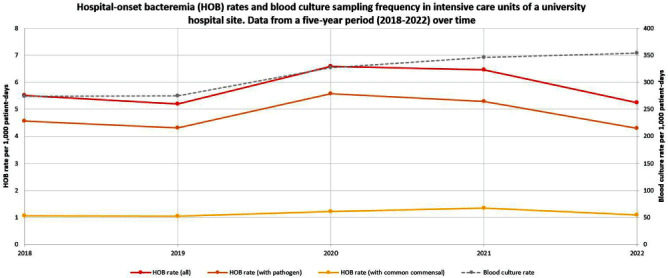# Advancing hospital infection surveillance: Automated detection of hospital-onset bacteremia in a large university hospital

**DOI:** 10.1017/ash.2024.321

**Published:** 2024-09-16

**Authors:** Seven Johannes Sam Aghdassi, Ferenc Darius Rüther, Christine Geffers, Luis Alberto Peña Diaz, Michael Behnke

**Affiliations:** Charité – Universitätsmedizin Berlin, corporate member of Freie Universität Berlin and Humboldt-Universität zu Berlin, Institute of Hygiene and Environmental Medicine; Charité – Universitätsmedizin Berlin, corporate member of Freie Universität Berlin and Humboldt-Universität zu Berlin

## Abstract

**Background:** Traditional manual surveillance of healthcare-associated infections often neglects certain hospital areas. This study investigated the potential of automated hospital-onset bacteremia (HOB) detection to enhance surveillance comprehensiveness. Our focus was on assessing baseline HOB levels across a large university hospital and demonstrating the algorithm’s ability to discern infection patterns in different hospital areas. **Method:** Conducted at one of the three sites of Charité university hospital in Berlin, Germany, our study employed an automated HOB detection algorithm, collaboratively developed with the PRAISE (Providing a Roadmap for Automated Infection Surveillance in Europe) network. HOB was defined as a positive blood culture (BC) with a recognized pathogen or two positive BC with common commensals, occurring two days post-admission or later. A retrospective data analysis spanned 2018-2022, with data extraction in January 2024. HOB rates were calculated per 1,000 patient-days and correlated with BC sampling frequency. Trends over time were examined. A comparison was made between intensive care units (ICU) and non-ICU. **Result:** Data from 58 wards (10 ICU, 48 non-ICU) with 262,058 in-patient admissions and 1,634,793 patient-days were included. Baseline characteristics of the hospital site are summarized in Table 1. Aggregated HOB rates over a 5-year period (Table 2) were substantially higher in ICU compared to non-ICU, applicable to all HOB, HOB with common commensals, and HOB with pathogens. A scatterplot (Figure 1) illustrates a strong correlation between BC sampling frequency and HOB rates in ICU, while Figure 2 displays HOB rates and BC sampling frequency in ICU over time. Notably, HOB rates were higher in the years 2020 and 2021, followed by a decrease in 2022, despite a continually high BC sampling rate. Discussion: The algorithm consistently detected HOB, providing insights into distinctions between ICU and non-ICU. While fewer HOB occurred in non-ICU, continuous monitoring remains crucial, given that the total number of HOB in non-ICU was higher than in ICU, and HOB in non-ICU might currently be overlooked by established surveillance activities. The automated system also demonstrated its effectiveness in identifying trends over time. Although speculative, it appears likely that the higher HOB rates in 2020 and 2021 were at least partially attributable to the COVID-19 pandemic and accompanying factors. There was a notable correlation between HOB and BC sampling frequency. However, the fact that HOB incidence in ICU decreased in 2022 despite persistently high BC sampling frequency rates, indicates the influence of other factors on HOB rates.

**Figure:**
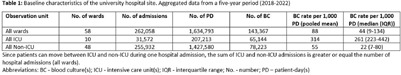

**Figure:**
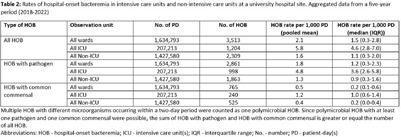

**Figure:**
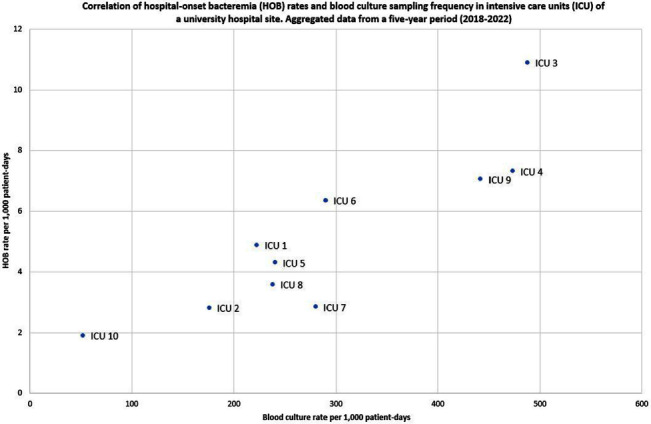

**Figure:**